# Barnacles Mating Optimizer with Deep Transfer Learning Enabled Biomedical Malaria Parasite Detection and Classification

**DOI:** 10.1155/2022/7776319

**Published:** 2022-06-01

**Authors:** Ashit Kumar Dutta, R. Uma Mageswari, A. Gayathri, J. Mary Dallfin Bruxella, Mohamad Khairi Ishak, Samih M. Mostafa, Habib Hamam

**Affiliations:** ^1^Department of Computer Science and Information System, College of Applied Sciences, AlMaarefa University, Riyadh 11597, Saudi Arabia; ^2^Department of Computer Science and Engineering, Vardhaman College of Engineering (Autonomous), Hyderabad, Telangana, India; ^3^Department of Information Technology, School of Information Technology and Engineering, Vellore Institute of Technology, Vellore, India; ^4^Department of Computer Science and Information Technology, Kalasalingam Academy of Research and Education, Krishnankoil, India; ^5^School of Electrical and Electronic Engineering, Universiti Sains Malaysia, Nibong Tebal 14300, Pulau Pinang, Malaysia; ^6^Faculty of Computers and Information, South Valley University, Egypt; ^7^Faculty of Engineering, Université de Moncton, Moncton, NB E1A 3E9, Canada; ^8^School of Electrical Engineering, Department of Electrical and Electronic Engineering Science, University of Johannesburg, Johannesburg 2006, South Africa

## Abstract

Biomedical engineering involves ideologies and problem-solving methods of engineering to biology and medicine. Malaria is a life-threatening illness, which has gained significant attention among researchers. Since the manual diagnosis of malaria in a clinical setting is tedious, automated tools based on computational intelligence (CI) tools have gained considerable interest. Though earlier studies were focused on the handcrafted features, the diagnostic accuracy can be boosted through deep learning (DL) methods. This study introduces a new Barnacles Mating Optimizer with Deep Transfer Learning Enabled Biomedical Malaria Parasite Detection and Classification (BMODTL-BMPC) model. The presented BMODTL-BMPC model involves the design of intelligent models for the recognition and classification of malaria parasites. Initially, the Gaussian filtering (GF) approach is employed to eradicate noise in blood smear images. Then, Graph cuts (GC) segmentation technique is applied to determine the affected regions in the blood smear images. Moreover, the barnacles mating optimizer (BMO) algorithm with the NasNetLarge model is employed for the feature extraction process. Furthermore, the extreme learning machine (ELM) classification model is employed for the identification and classification of malaria parasites. To assure the enhanced outcomes of the BMODTL-BMPC technique, a wide-ranging experimentation analysis is performed using a benchmark dataset. The experimental results show that the BMODTL-BMPC technique outperforms other recent approaches.

## 1. Introduction

Biomedical engineering turned out to be helpful for decision-making in the healthcare sector [1, 2]. It is obvious throughout health care, from analysis and diagnosis to recovery and treatment, and entered the social conscience through the proliferation of implanted health care devices, namely, artificial hips and pacemakers, for further futuristic techniques like 3D printing of biological organs and stem cell engineering. Biomedical engineering focuses on the advancement that improves healthcare and human health at each level. Malaria, a deadly disease caused by Plasmodium parasites [3], is still a serious health concern around the globe, particularly in third world countries. Malaria can be prevented and is curable when proper methods and initiatives are efficiently deployed that mainly depend on earlier diagnoses of malarial parasites [4]. In the literature, several approaches, namely, microscopic diagnoses, clinical diagnoses, polymerase chain reaction (PCR), and rapid diagnostic test (RDT) have been reported that identify malarial parasites in a patient [5].

Traditional diagnosis techniques such as PCR and clinical diagnosis are conducted in research communities, and the accuracy and efficiency depend largely on the level of human experts [6]. These experts are present inadequately in unreached remote areas whereby malaria could be more prominent. RDT and microscopic diagnoses are two highly effective malaria diagnosis approaches that make a huge contribution to malaria control [7]. RDT is an efficient diagnosis method as it does not need any microscope or trained professional and offers diagnosis within fifteen minutes. The World Health Organization stated the RDT suffers from several limitations such as lack of insensitivity, expensive, and vulnearable to damage. The microscopic system does not suffer from this shortcoming and it is considered to be efficient for malaria parasite diagnosis [8]. However, these techniques require the existence of a skilled microscopist [9]. Automated microscopic malaria parasite diagnosis includes segmentation of cells and classification of infected cells, and the acquisition of the microscopic blood smear images could be a powerful diagnosis method [10]. It has been noticed that effective identification of malaria parasites and segmentation of blood cells are utilized to implement counting. Cell segmentation is a well-studied area and better outcomes have already been demonstrated in various research studies [11]. Therefore, machine learning (ML)-based automated tools have been developed to resolve the limitations.

Existing image analysis enabled computer-aided diagnosis (CAD) models through the use of machine learning (ML) approaches employed to blood smear images. On the other hand, convolutional neural networks (CNN), a kind of deep learning (DL) model, offers high scalability and effective outcome. The CNN is commonly employed for extracting detailed information via weights and pooling. But the training data size considerably influences the classifier results compared to conventional ML models. For resolving this issue, transfer learning models can be modelled where the features are derived from the pretrained models. Earlier works that are focused on the identification of infected cells involve tools and techniques from image processing, computer vision, and machine learning. However, solutions for the classification of infected cells, which are both accurate and computationally efficient, have not been studied to the best of our knowledge.

This study introduces a new Barnacles Mating Optimizer with Deep Transfer Learning Enabled Biomedical Malaria Parasite Detection and Classification (BMODTL-BMPC) model. The presented BMODTL-BMPC model involves the Gaussian filtering (GF) approach employed to eradicate noise in blood smear images. In addition, the Graph cut (GC) segmentation technique is applied to determine the affected regions in the blood smear images. Besides, the barnacles mating optimizer (BMO) algorithm with the NasNetLarge model is employed for the feature extraction process. Finally, the extreme learning machine (ELM) classification model is employed for the identification and classification of malaria parasites. To assure improvised outcomes of the BMODTL-BMPC technique, a wide-ranging experimentation analysis is performed using a benchmark dataset. The following are the key contributions of our work:

- It proposes a new biomedical intelligent technique for the recognition and grouping of malaria parasites on blood smear images. This technique englobes GF-based preprocessing, GC segmentation, NasNetLarge feature extraction, BMO-based hyperparameter optimization, and ELM classification.
- A wide-ranging experimentation analysis is carried out using a benchmark dataset.
- The experimental results demonstrated the significance of the BMODTL-BMPC technique over other approaches.

The remainder of this paper is organized as follows.  Section 2 discusses the most significant works in relation to our subject.  Section 3 illustrates the details of the proposed methods and materials.  Section 4 presents experimental details, results, and discussion. Concluding remarks are given in Section 5.

## 2. Related Works

Recent neural network-based techniques, including machine learning and deep learning, have been intensively used in the healthcare and biomedical engineering sector [12–14]. Meng et al. [14] present a new NCGCN with CNN feature extraction, neighbor correlation mining, and graph depiction elements. This technique initially extracts CNN representation in each parasite image and next creates the neighbor correlation amongst CNN feature with integrating KNN and radius graph creating techniques, with effective GCN on CNN feature and its correlation. Fuhad et al. [15] present a totally automated CNN-based method for the analysis of malaria in the microscopic blood smear image. The variation of approaches containing skill distillation, data expansion, autoencoder, feature extracting by CNN technique, and classification by SVM or KNN can be implemented in 3 trained processes.

In [16], a DL-based approach (named DeepSweep) is conceived to train on the haplotypic image in a genetic region with identified sweeps for identifying loci under positive selection. The DL method detects positive selective signatures from malaria parasite WGS data. Moreover, this technique was generalizable; it is trained for detecting other kinds of selection. Maqsood et al. [17] present the customized CNN technique, which demonstrates every observed DL technique. It uses the bilateral filtering (BF) and image augmentation approach to highlight features of red blood cells before training the method. The utilization of image augmentation approaches can avoid overfitting and achieves generalization.

The authors in [18] introduced a new hybrid method dubbed RAL-CNN-SVM for malaria classification. All the RAL-CNN blocks have residual learning and novel attention progress that is mostly utilized for extracting image depth activation features. The classification layer gets the benefit of a strong point of the SVM classifier technique. Oyewola et al. [19] present a new DL technique named data augmentation CNN (DACNN), trained by reinforcement learning (RL) for tackling this issue. The performance of the presented DACNN technique is related to CNN and directed acyclic graph CNN (DAGCNN) techniques.

## 3. Materials and Methods

**Figure 1:**
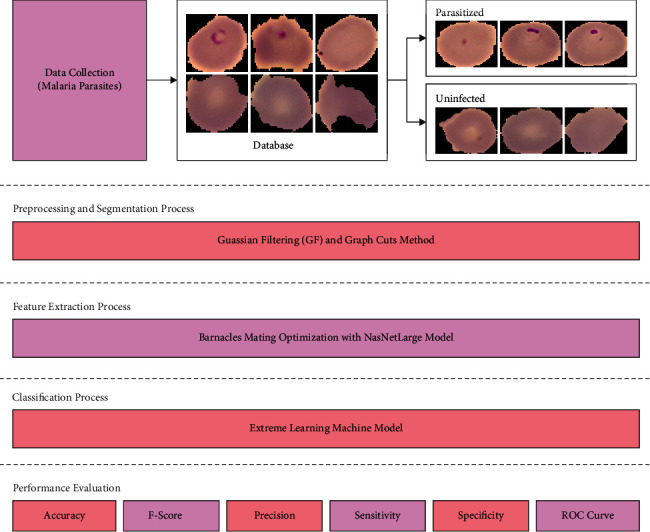
Block diagram of the BMODTL-BMPC model.

In this study, a new BMODTL-BMPC technique has been designed for intelligent recognition and classification of malaria parasites on blood smear images. The presented BMODTL-BMPC technique encompasses GF-based preprocessing, GC segmentation, NasNetLarge feature extraction, BMO-based hyperparameter optimization, and ELM classification. The workflow of the BMODTL-BMPC model is shown in Figure 1. Initially, the preprocessing and segmentation processes are carried out using the GF technique and GC technique, respectively. Then, the segmented images are fed into the BMO-NasNetLarge model to extract feature vectors. Finally, they are entered into the ELM model for the classification of malaria parasites.

### 3.1. Preprocessing: GF Technique

At the first level, the GF technique can be utilized to eradicate noise in blood smear images. Firstly, the GF approach can be employed for removing the noise and boosting the medical images. It is commonly utilized to remove noise and smooth images [20]. It necessitates massive computing resources. Here, the convolution operator can be defined by the Gaussian operator, and the recommendation of Gaussian smoothening can be attained by the use of convolution. The Gaussian operators in 1D can be defined as follows:

(1) $$\begin{array}{c} G_{1D}\left(x\right)=\frac{1}{\sqrt{2\pi }\sigma }e^{-\left(x^{2}/2\sigma^{2}\right)}. \end{array}$$

The optimum smoothening filter for the image is localized in frequency as well as spatial domains, where the ambiguity relationship can be satisfied using the following equation [20]:

(2) $$\begin{array}{c} \Delta x\Delta \omega \geq \frac{1}{2}. \end{array}$$

The Gaussian operators in 2D are represented by the use of the following equation:

(3) $$\begin{array}{c} G_{2D}\left(x,y\right)=\frac{1}{2\pi \sigma^{2}}e^{-\left(x^{2}+y^{2}/2\sigma^{2}\right)}, \end{array}$$

where *σ*  (sigma) implies the standard deviation (SD) of the Gaussian operators. When it holds higher values, the smoothening is higher and (*x*,  *y*) represents the Cartesian coordinates of the image.

### 3.2. Graph Cuts Segmentation

Once blood smear images are preprocessed, the next stage is to perform GC segmentation to determine the affected regions in the blood smear images. The GC-based segmentation model lies in the extraction of diseased regions from the target image with detailed information [21]. It is widely used to segment medical images due to its benefit of attaining global optimum solutions. Here, the GC segmentation can be considered as the energy function minimization problem as given in the following equation:

(4) $$\begin{array}{c} E\left(f\right)=\left(1-\lambda \right)\sum_{u\in P}R\left(f_{u}\right)+\lambda \sum_{u\in P,v\in N_{u}}B\left(f_{u},f_{v}\right), \end{array}$$

where *P* implies pixel set of image *f*, *N*_*u*_ denotes 4‐neighboring pixel *u*, *R*(*f*_*u*_) indicates region term punishing individual pixels allocated to objects and backdrop, and *B*(*f*_*u*_, *f*_*v*_) indicates boundary component demanding a disjointedness between *u* and *v*. Here, the improved data produced via nonlinear mapping and the gradient data attained from the actual ROC are employed for the region and boundary term computation.

(5) $$\begin{array}{c} R\left(f_{u}\right)=\left\{\begin{array}{cc} 1-H\left(l_{u}\right), & {\text{iff}}_{u}=\text{abnormal”,}\\ H\left(l_{u}\right), & {\text{iff}}_{u}=\text{normal”,} \end{array}\right.\\ B\left(f_{u}f_{v}\right)=\left\{\begin{array}{cc} \text{ exp}\left(-\frac{{\left(l_{u}-l_{v}\right)}^{2}}{2\eta^{2}}\right)\cdot \frac{1}{d\left(u,v\right)}, & {\text{iff}}_{u}\neq f_{v},\\ 0, & {\text{iff}}_{u}=f_{v}, \end{array}\right. \end{array}$$

where *I*_*u*_ represents pixel intensities *u*, *d*(*u*,  *v*) indicates the spatial distance from *u* to *v*, and *η* offers the standard deviation of the variances designed by a pair of neighboring pixels in image *f* represented by the following equation:

(6) $$\begin{array}{c} \eta =\sqrt{\frac{1}{T_{u}}\sum_{u\in Pv\in N_{u}}{\left|l_{u}-l_{v}\right|}^{2}}, \end{array}$$

where *T*_*u*_ signifies a pixel number of set *P*. At energy function of GC, the weight *λ* computes the contribution of the boundaries and region components. If the value of *λ* is low, the region component acts as a main part in the GC.

### 3.3. Feature Extraction Using the Optimal NASNetLarge Model

For extracting feature vectors, the BMO algorithm with the NasNetLarge model is employed. The NASNetLarge method encompasses of encoder and decoder that is followed by a classification layer. There are two considerable variations in this method compared to Segnet that apply the pretrained VGG16 architecture for the encoder. The NasnetLarge-decoder net employs the initial 414 layers of NasnetLarge net (that is a well-trained network for ImageNet classification) as the encoder for decomposing images [22]. Then, select the first 414 layers since the size of the last layer is closer to the size of the original image; hence, it will not lack more data. Then, the pretrained weight is not employed but retrain the net through new information to fit NasnetLarge in this study because data are distinct from ImageNet.

Additionally, the decoder is distinct and also there are no pooling indices because NasnetLarge net produces complete data for the decoder. A suitable decoder could upsample its input feature map through the max-pooling layer. It comprises four blocks in the decoder. Each starts with upsampling that could extend the feature maps and convolutional and rectified linear units. Then, a batch normalization layer is employed to this map. The initial decoder that is closer to the final encoder might generate a multichannel feature map. This is analogous to how Segnet could produce a distinct number of channels and sizes as encoder input. The last output of the final decoder layer is passed to trainable softmax classification that generates K channel image of probability in which K denotes the class count. The forecasted segmentation corresponds to the class with the maximal possibility at all the pixels.

To appropriately tune the hyperparameters that exist in the NasNetLarge model, the BMO algorithm has been employed. The barnacles are microbes, which developed close to objects from the water. The barnacles are a long penis, and it endures mate with all the neighbors and competitors in the influence of its penis. The BMO technique was inspired by the mating process of barnacles [23]. Initially, the candidate solution is assumed as the barnacle, and the population initialization takes place using equation (7). The validation of the population and storage process occurs by locating the global solution obtained at the top of *X*. Afterward, the parent that mated is selected utilizing (8) and (9):

(7) $$\begin{array}{c} X=\left[\begin{array}{ccc} x_{1}^{1} & \cdots & x_{1}^{n}\\ \vdots & \ddots & \vdots \\ x_{N}^{1} & \cdots & x_{N}^{n} \end{array}\right], \end{array}$$

(8) $$\begin{array}{c} {\text{barnacle}}_{-}d=\text{randperm}\left(N\right), \end{array}$$

(9) $$\begin{array}{c} {\text{barnacle}}_{-}m=\text{randperm}\left(N\right), \end{array}$$

where *N* represents the entire barnacle population, *n* implies the control variable count, and barnacl_*d*  and barnacle_−_*m* denote the mating parents. While there is no specific formula in deriving the reproduction procedure of the barnacles, the BMO technique is emphasizing the genotype frequency of parents from producing the offspring based on the Hardy–Weinberg rule. It is noticeable the length of penis (*pl*) performs as an important part in determining the exploitation and exploration procedure. If the selection of barnacles endures mate from the restrict *pl* of Dad Barnacles, the exploitation process occurs. The following formula has been projected to produce a novel parameter of offspring in the barnacle parent:

(10) $$\begin{array}{c} x_{i}^{N_{-}\text{new}}=px_{{\text{barnacle}}_{-}d}^{N}+qx_{{\text{barnacle}}_{-}m}^{N}, \end{array}$$

where *p* denotes the arbitrary number from the uniform distribution of zero and one *q*=(1 − *p*), *x*_barnacle_−_*d*_^*N*^ and *x*_barnacle_−_*m*_^*N*^ refer to the parameters of Dad and Mum barnacles that are selected, and *p* and *q* signify the genotype frequency of Dad and Mum barnacles from the novel offspring. Afterward, the exploration process is provided as follows:

(11) $$\begin{array}{c} x_{i}^{n_{-}\text{new}}=\text{rand}\left(\right)\times x_{barnacle_{-}m}^{n}, \end{array}$$

where rand() refers to the arbitrary number between zero and one. In the above formula, it is defined that the recently generated offspring in the mother barnacle reaches the sperms released by other barnacles. In the iteration procedure, the place of barnacles is upgraded. Finally, the BMO is signified by the approximation of global optimal to the optimized problem.

The BMO approach mainly determines a fitness value with the goal of attaining maximum classifier results. It computes a positive integer in order to demonstrate improved outcomes on the candidate solution. Here, reducing the classifier error rate can be treated as the fitness function, as given in equation (12). The optimum solution holds the least error rate and the poorly attained solution offers high error rate.

(12) $$\begin{array}{c} \text{fitness}\left(x_{i}\right)=\text{Classifier Error Rate}\left(x_{i}\right),\\ =\frac{\text{number of misclassified samples}}{\text{total number of samples}}*100. \end{array}$$

### 3.4. ELM Classification

**Figure 2:**
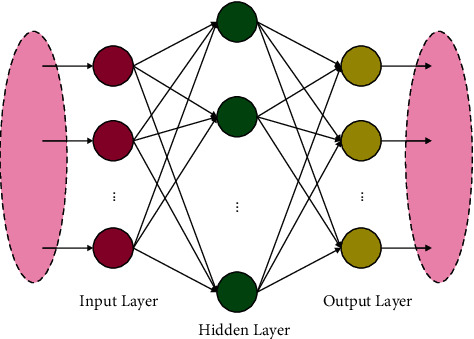
Structure of ELM.

In the final stage, the ELM classification model is employed for the identification and classification of malaria parasites. It is one of the popular kinds of artificial neural networks that has gained considerable interest in the past few decades [24]. The ELM network provides a hybrid template with a higher divergence of feature transmission that is applied in the hidden layer. This could be directly in regression and multiclass classification. The ELM determines a learning methodology for Single Hidden Neural Network (SHNN) with random initialization of inputted bias and weight and the systematic calculation of the output weight. The major arrangement of ELM-NN has been demonstrated in Figure 2. In the ELM network, assume *M* training instances and *D* dimension, as follows:

(13) $$\begin{array}{c} \left(x^{\left(n\right)},t^{\left(n\right)}\right),n=1:Μ, \end{array}$$

where *t*^(*n*)^ ∈ ℝ^*K*^ and *x*^(*n*)^ ∈ ℝ^*D*^. A feedforward neural network-based ELM concept is given as follows:

(14) $$\begin{array}{c} \sum_{m=1}^{N}\beta_{m}h\left(w_{m}^{T}X^{\left(n\right)}+b_{m}\right)=t^{\left(n\right)}. \end{array}$$

Now, *h* indicates the activation function, *b*_*m*_ defines the bias for *m*^*th*^ hidden layer, and*N* determines the hidden layers.

*w*_*m*_=[*w*_*m*1_, *w*_*m*2_,…*w*_*mD*_] denotes the inputted weight vector that joints the inputted neuron to the *m*^*th*^ neuron of the invisible layer and *β*_*m*_=[*β*_*m*1_,  *β*_*m*2_,…, *β*_*mK*_] defines the vector of weight that joints the *m*^*𝔠h*^ neuron of the invisible layer to the outputted layer. It can be briefly explained in the following equation:

(15) $$\begin{array}{c} H\beta =T. \end{array}$$

Now,

(16) $$\begin{array}{c} H=\left\{\begin{array}{ccc} g\left(w_{m}^{T}X^{\left(1\right)}+b_{1}\right) & \cdots & g\left(w_{M}^{T}X^{\left(1\right)}+b_{1}\right)\\ \vdots & \ddots & \vdots \\ g\left(w_{1}^{T}X^{\left(N\right)}+b_{1}\right) & \cdots & g\left(w_{M}^{T}X^{\left(N\right)}+b_{M}\right) \end{array}\right\},\\ H={\left[\beta_{1}^{T},\beta_{2}^{T},\ldots ,\beta_{M}^{T}\right]}_{M\times N}^{T}T={\left[t_{1}^{T},\;t_{2}^{T},\ldots ,t_{M}^{T}\right]}_{N\times K}^{T}. \end{array}$$

Further, the hidden neuron has fewer numbers when compared to the training instance and *H* represents a nonsquare matrix. Therefore, the subsequent formula is used to resolve the shortcomings:

(17) $$\begin{array}{c} \hat{\beta }=H^{↑}T, \end{array}$$

where *H*† defines the generalized Moore–Penrose matrix inverse.

## 4. Experimental Results

**Figure 3:**
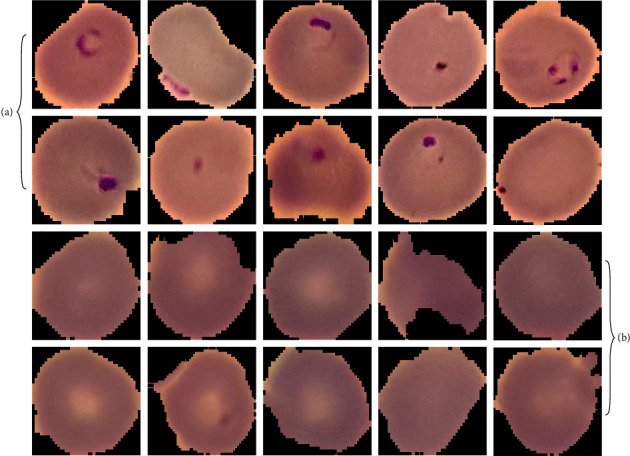
Sample images of (a) parasitized class and (b) uninfected class.

In this section, the malaria parasite classification results of the BMODTL-BMPC model on blood smear images are carried out. The results are tested using the malaria dataset comprising a set of 26161 samples [25]. It includes 1312 samples under parasitized class and 13029 samples under uninfected class.  Figure 3 displays a sample set of blood smear images. The proposed model is simulated using the Python 3.6.5 tool. The results are investigated under distinct sizes of training (TR) and testing (TS) data, as given below:

- TR/TS_data of 90 : 10
- TR/TS_data of 80 : 20
- TR/TS_data of 70 : 30, and
- TR/TS_data of 60 : 40

**Figure 4:**
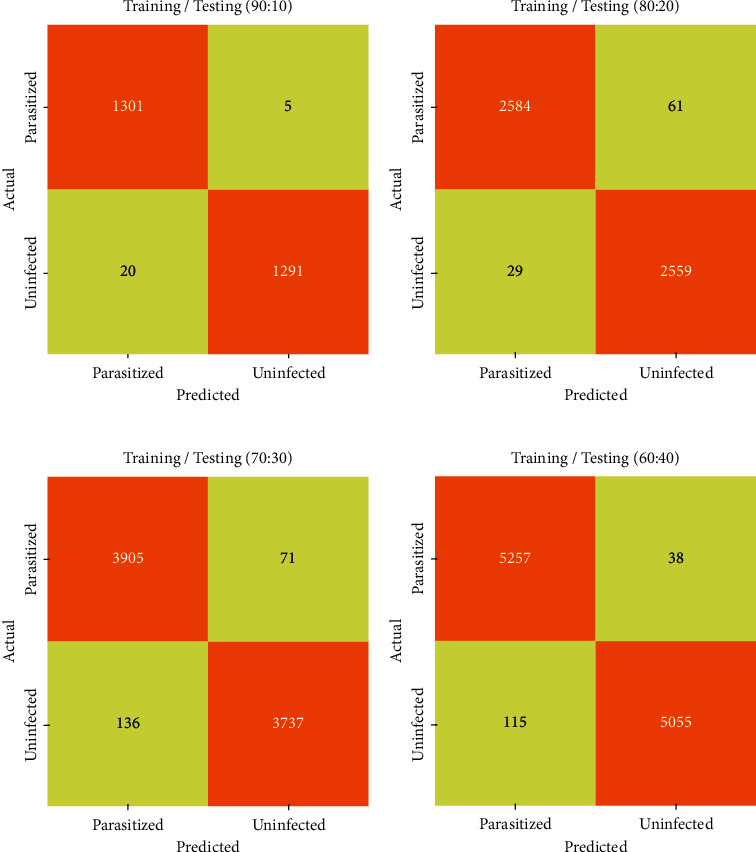
Confusion matrices of the BMODTL-BMPC model: (a) TR/TS_data of 90 : 10; (b) TR/TS_data of 80 : 20; (c) TR/TS_data of 70 : 30; (d) TR/TS_data of 60 : 40.

The confusion matrices offered by the BMODTL-BMPC model on malaria parasite classification outcomes on distinct sizes of TR/TS_dataset are given in Figure 4. With TR/TS_data of 90 : 10, the BMODTL-BMPC model has recognized 1301 samples in parasitized class and 1291 samples in uninfected class. In addition, with TR/TS_data of 80 : 20, the BMODTL-BMPC model has accepted 2584 samples in parasitized class and 2559 samples in uninfected class. Moreover, with TR/TS_data of 70 : 30, the BMODTL-BMPC model has recognized 3905 samples into parasitized class and 3737 samples into uninfected class. Finally, with TR/TS_data of 60 : 40, the BMODTL-BMPC model has acknowledged 5257 samples into parasitized class and 5055 samples into uninfected class.

**Figure 5:**
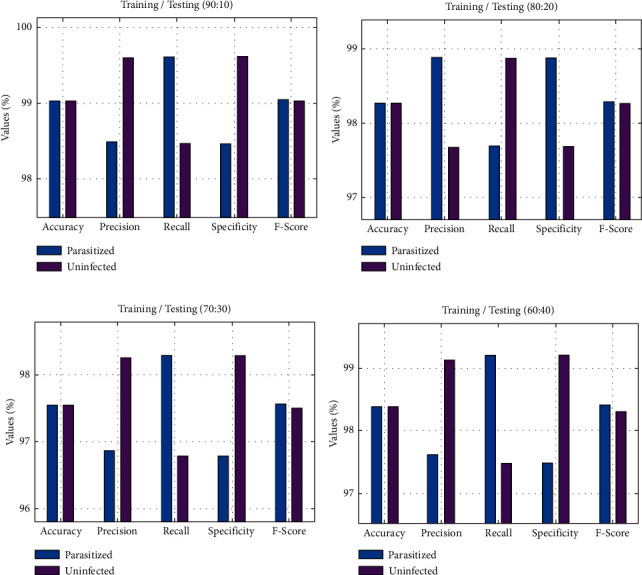
Malaria parasite classification outcome of the BMODTL-BMPC model: (a) TR/TS_data of 90 : 10; (b) TR/TS_data of 80 : 20; (c) TR/TS_data of 70 : 30; (d) TR/TS_data of 60 : 40.

Table 1 and Figure 5 demonstrate the overall malaria parasite classification outcomes of the BMODTL-BMPC model with distinct sizes of training/testing data (TR/TS_data). With TR/TS_data of 90 : 10, the BMODTL-BMPC model has classified parasitized samples with accu_*y*_ of 99.04%, prec_*n*_ of 99.05%, reca_*l*_ of 99.05%, spec_*y*_ of 99.05%, and *F*_score_ of 99.04%. Besides, the BMODTL-BMPC model has categorized uninfected samples with accu_*y*_ of 99.04%, prec_*n*_ of 99.61%, reca_*l*_ of 98.47%, spec_*y*_ of 99.62%, and *F*_score_ of 99.04%. Likewise, with TR/TS_data of 60 : 40, the BMODTL-BMPC model has categorized parasitized samples with accu_*y*_ of 98.54%, prec_*n*_ of 97.86%, reca_*l*_ of 99.28%, spec_*y*_ of 97.78%, and *F*_score_ of 98.57%. Besides, the BMODTL-BMPC model has considered uninfected samples with accu_*y*_ of 98.54%, prec_*n*_ of 99.25%, reca_*l*_ of 97.78%, spec_*y*_ of 99.28%, and *F*_score_ of 98.51%.

**Figure 6:**
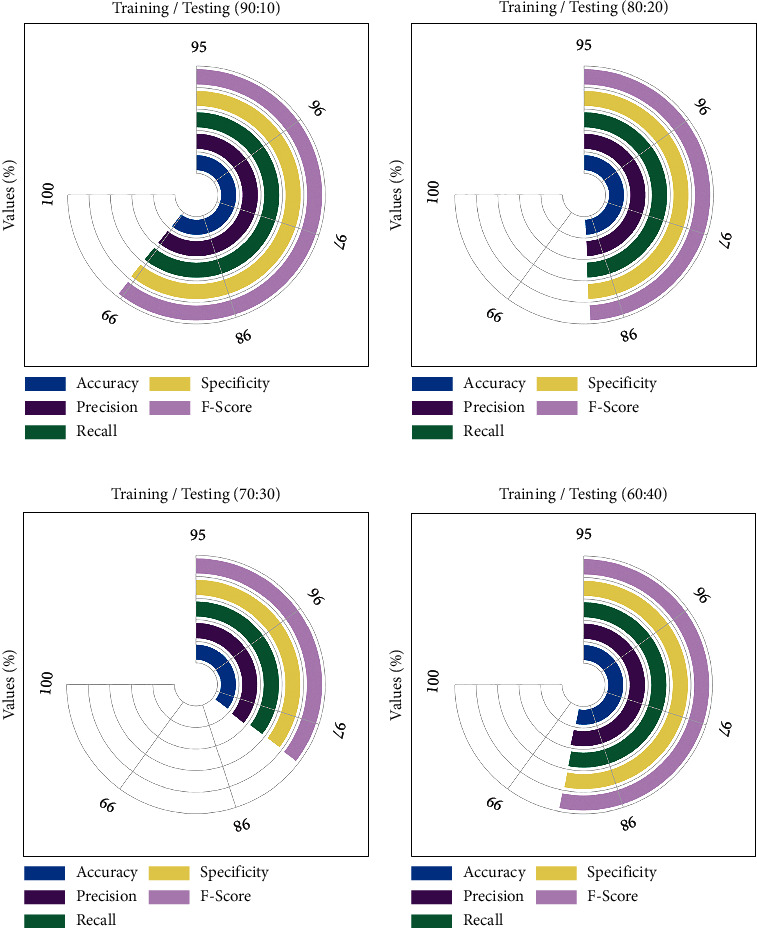
Average malaria parasite classification outcome of BMODTL-BMPC model: (a) TR/TS_data of 90 : 10; (b) TR/TS_data of 80 : 20; (c) TR/TS_data of 70 : 30; (d) TR/TS_data of 60 : 40.

Figure 6 depicts an average malaria parasite classification outcome of the BMODTL-BMPC model with distinct sizes of TR/TS_dataset. On TR/TS_data of 90 : 10, the BMODTL-BMPC model has offered average accu_*y*_ of 99.04%, prec_*n*_ of 99.05%, reca_*l*_ of 99.05%, spec_*y*_ of 99.05%, and *F*_score_ of 99.04%. Moreover, on TR/TS_data of 80 : 20, the BMODTL-BMPC model has provided average accu_*y*_ of 98.28%, prec_*n*_ of 98.28%, reca_*l*_ of 98.29%, spec_*y*_ of 98.29%, and *F*_score_ of 98.28%. Furthermore, on TR/TS_data of 70 : 30, the BMODTL-BMPC model has attained average accu_*y*_ of 97.36%, prec_*n*_ of 97.39%, reca_*l*_ of 97.35%, spec_*y*_ of 97.35%, and *F*_score_ of 97.36%. At the same time, on TR/TS_data of 60 : 40, the BMODTL-BMPC model has exhibited average accu_*y*_ of 98.54%, prec_*n*_ of 98.56%, reca_*l*_ of 98.53%, spec_*y*_ of 98.53%, and *F*_score_ of 98.54%.

**Figure 7:**
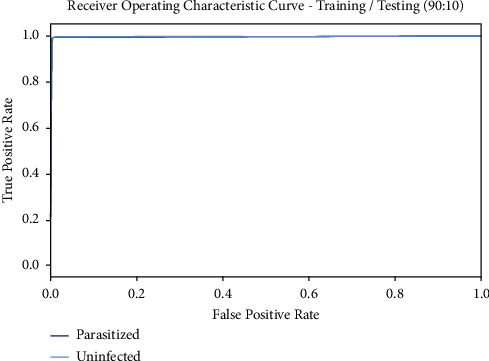
ROC curve of the BMODTL-BMPC model with TR/TS_data of 90 : 10.

Figure 7 illustrates the ROC curve obtained by the BMODTL-BMPC model on the TR/TS_data of 90 : 10. The figure indicated that the BMODTL-BMPC model has shown effectual malaria parasite classification results. The BMODTL-BMPC model has gained maximum ROC values on the classification of two class labels, namely, parasitized and uninfected.

**Figure 8:**
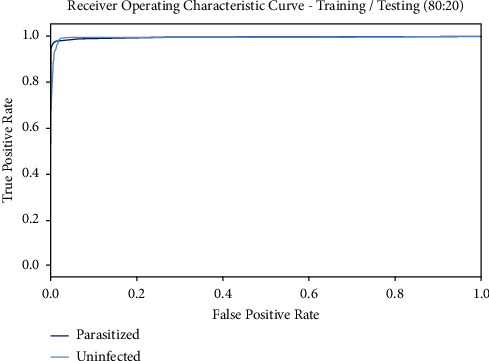
ROC curve of the BMODTL-BMPC model with TR/TS_data of 90 : 10.

Figure 8 exemplifies the ROC curve gotten by the BMODTL-BMPC model on the TR/TS_data of 80 : 20. The figure designated that the BMODTL-BMPC model has exposed capable malaria parasite classification results. The BMODTL-BMPC model has expanded supreme ROC values on the classification of two class labels, specifically parasitized and uninfected.

**Figure 9:**
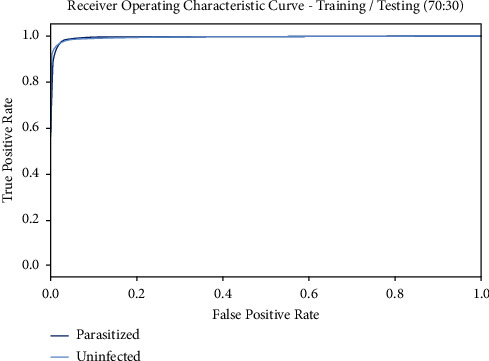
ROC curve of the BMODTL-BMPC model with TR/TS_data of 90 : 10.

Figure 9 demonstrates the ROC curve gained by the BMODTL-BMPC model on the TR/TS_data of 70 : 30. The figure specified that the BMODTL-BMPC model has revealed effective malaria parasite classification results. The BMODTL-BMPC model has gained maximum ROC values on the classification of two class labels, namely, parasitized and uninfected.

**Figure 10:**
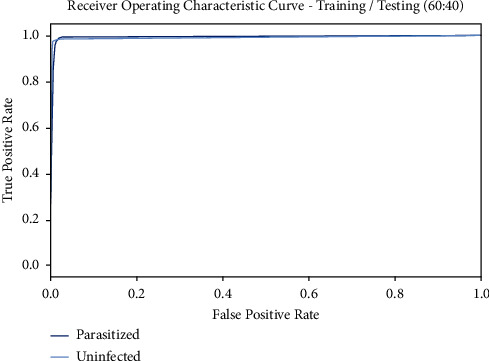
ROC curve of the BMODTL-BMPC model with TR/TS_data of 90 : 10.

Figure 10 displays the ROC curve attained by the BMODTL-BMPC model on the TR/TS_data of 60 : 40. The figure showed that the BMODTL-BMPC model has revealed capable malaria parasite classification results. The BMODTL-BMPC model has increased ROC values in the classification of two class labels, namely, parasitized and uninfected.

**Figure 11:**
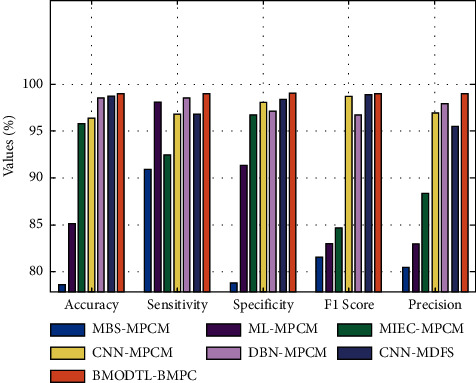
Comparative analysis of the BMODTL-BMPC model with recent methods.

Table 2 and Figure 11 illustrate an extensive comparative study of the BMODTL-BMPC approach with recent methods [15]. The obtained results indicated that the BMODTL-BMPC approach has resulted in maximum classification outcome over the other methods.

On examining the outcome in terms of accu_*y*_, the BMODTL-BMPC model has offered higher accu_*y*_ of 99.04% whereas the MBS-MPCM, ML-MPCM, MIEC-MPCM, CNN-MPCM, DBN-MPCM, and CNN-MDFS models have resulted in reduced accu_*y*_ of 78.59%, 85.14%, 95.80%, 96.34%, 98.51%, and 98.71%, respectively.

Similarly, on inspecting the outcome in terms of accu_*y*_, the BMODTL-BMPC model has presented increase sens_*y*_ of 99.05% whereas the MBS-MPCM, ML-MPCM, MIEC-MPCM, CNN-MPCM, DBN-MPCM, and CNN-MDFS models have reached slightly decreased sens_*y*_ of 90.94%, 98.08%, 92.49%, 96.78%, 98.54%, and 96.84%, respectively. After observing the results and discussion, it is verified that the BMODTL-BMPC model is a proficient tool for malaria parasite classification.

## 5. Conclusion

In this article, a new BMODTL-BMPC technique has been projected for the intelligent recognition and grouping of malaria parasites on blood smear images. The presented BMODTL-BMPC technique encompasses GF-based preprocessing, GC segmentation, NasNetLarge feature extraction, BMO-based hyperparameter optimization, and ELM classification. To appropriately tune the hyperparameters that exist in the NasNetLarge model, the BMO algorithm has been employed. In the final stage, the ELM classification model is employed for the identification and classification of malaria parasites. For ensuring the enhanced outcomes of the BMODTL-BMPC technique, a wide-ranging experimentation analysis is performed using a benchmark dataset. The experimental outcome highlighted the effectual outcomes of the BMODTL-BMPC technique over recent approaches. In the future, deep instance segmentation models can be employed to improve classification outcomes.

## Figures and Tables

**Table 1:** Overall malaria parasite classification outcome of the BMODTL-BMPC model.

Class labels	Accuracy (%)	Precision (%)	Recall (%)	Specificity (%)	F-score (%)
*Training/testing (90* *:* *10)*					
Parasitized	99.04	98.49	99.62	98.47	99.05
Uninfected	99.04	99.61	98.47	99.62	99.04
Average	**99.04**	**99.05**	**99.05**	**99.05**	**99.04**

*Training/testing (80:20)*					
Parasitized	98.28	98.89	97.69	98.88	98.29
Uninfected	98.28	97.67	98.88	97.69	98.27
Average	**98.28**	**98.28**	**98.29**	**98.29**	**98.28**

*Training/testing (70:30)*					
Parasitized	97.36	96.63	98.21	96.49	97.42
Uninfected	97.36	98.14	96.49	98.21	97.31
Average	**97.36**	**97.39**	**97.35**	**97.35**	**97.36**

*Training/testing (60:40)*					
Parasitized	98.54	97.86	99.28	97.78	98.57
Uninfected	98.54	99.25	97.78	99.28	98.51
Average	**98.54**	**98.56**	**98.53**	**98.53**	**98.54**

**Table 2:** Comparative malaria parasite classification outcome of the BMODTL-BMPC model with existing models [15].

Methods	Accuracy (%)	Sensitivity (%)	Specificity (%)	*F*1 score (%)	Precision (%)
MBS-MPCM	78.59	90.94	78.79	81.58	80.38
ML-MPCM	85.14	98.08	91.32	82.95	82.98
MIEC-MPCM	95.80	92.49	96.73	84.58	88.29
CNN-MPCM	96.34	96.78	98.02	98.71	96.94
DBN-MPCM	98.51	98.54	97.10	96.75	97.93
CNN-MDFS	98.71	96.84	98.38	98.94	95.49
BMODTL-BMPC	99.04	99.05	99.05	99.05	99.04

## Data Availability

The dataset used in this paper is publicly available via the following link: https://lhncbc.nlm.nih.gov/LHC-publications/pubs/MalariaDatasets.html.
